# Inhibition of IL-17 ameliorates systemic lupus erythematosus in Roquin^san/san^ mice through regulating the balance of TFH cells, GC B cells, Treg and Breg

**DOI:** 10.1038/s41598-019-41534-1

**Published:** 2019-03-26

**Authors:** Seon-yeong Lee, Seung Hoon Lee, Hyeon-Beom Seo, Jun-Geol Ryu, KyungAh Jung, Jeong Won Choi, JooYeon Jhun, Jin-Sil Park, Ji Ye Kwon, Seung-Ki Kwok, Jeehee Youn, Sung-Hwan Park, Mi-La Cho

**Affiliations:** 1 Rheumatism Research Center, Catholic Research Institute of Medical Science, College of Medicine, The Catholic University of Korea, Seoul, Republic of Korea; 2 Laboratory of Immune Network, Conversant Research Consortium in Immunologic disease, College of Medicine, The Catholic University of Korea, Seoul, Republic of Korea; 3 Impact Biotech, Korea 505 Banpo-Dong, Seocho-Ku, 137-040 Seoul Republic of Korea; 4 Division of Rheumatology, Department of Internal Medicine, Seoul St. Mary’s Hospital, College of Medicine, The Catholic University of Korea, Seoul, Republic of Korea; 5 Department of Biomedical Sciences, College of Medicine, Hanyang University, Seoul, South Korea; 6 Department of Medical Lifescience, College of Medicine, The Catholic University of Korea, 222, Banpo-daero, Seocho-gu, Seoul 06591 Republic of Korea; 7 Department of Biomedicine & Health Sciences, College of Medicine, The Catholic University of Korea, 222, Banpo-daero, Seocho-gu, Seoul 06591 Republic of Korea; 8 Department of Immunology, Blavatnik Institute, Harvard Medical School, Boston, MA USA

## Abstract

Systemic lupus erythematosus (SLE) is mediated by a chronic and dysregulated inflammatory response. Interleukin (IL)-17, a proinflammatory cytokine, and T helper (Th)17 cells are associated with chronic autoimmune diseases. We hypothesized that inhibition of IL-17 would decrease the numbers of T cell subsets that function as B-cell helpers, as well as B-cell differentiation into plasma cells and autoantibody expression. The IL-17 level was increased markedly in Roquin^san/san^ mice. Loss of IL-17 in Roquin^san/san^ mice improved nephritis by downregulating immunoglobulin (Ig)G, IgG1, and IgG2a production. Formation of germinal centers (GCs), and follicular B- and T-cell differentiation was reduced, whereas the number of regulatory T (Treg) cells and immature B cells was increased, by IL-17 deficiency in Roquin^san/san^ mice. These results suggest that IL-17 inhibition can ameliorate SLE by inhibiting B-cell differentiation into GCs. Therefore, IL-17–producing Th17 cells show promise as a target for development of novel therapeutics for SLE.

## Introduction

Systemic lupus erythematosus (SLE) is a systemic autoimmune disease mediated by a chronic and excessive inflammatory response^1^. Damage to multiple organs results from the dysregulated immune inflammatory response mediated by autoantibodies (autoAbs) and immune complexes in SLE. For example, nephritis occurs in approximately 50% of SLE patients and induces premature death^2–5^.

Proinflammatory cytokines contribute to the pathogenesis of SLE. Indeed, serum levels of proinflammatory cytokines, such as interleukin (IL-1β, IL-6, and tumor necrosis factor (TNF)-α are correlated with SLE activity^6^. IL-17 is a proinflammatory cytokine involved in the development of several autoimmune diseases, including SLE. The serum level of IL-17A and numbers of IL-17–producing T cells were increased both in patients with SLE and in a mouse model of lupus^7,8^. In addition, the number of IL-17–producing T cells in peripheral blood is increased in SLE patients^9,10^, and an elevated serum IL-17 level is correlated with SLE progression^10^.

In SLE patients, the population of T follicular helper (Tfh) cells, which play a key role in B-cell differentiation into plasma cells in the germinal centers (GCs), as well as autoAb production, are increased^11,12^. IL-17 is associated with Tfh, GCs, and autoAbs. Indeed, autoAb overproduction is reportedly caused by IL-17 stimulation of peripheral blood mononuclear cells from patients with lupus nephritis^13^. Moreover, IL-17 activates B cells and promotes formation of GCs^14^, as do IL-17–producing Tfh cells^15^.

Roquin was identified as a CCCH-type zinc finger protein and diminish abnormal inducible T cell co-stimulator (ICOS) expression on T cells^16,17^. It has been demonsrated that Roquin deficiency leads to autoimmunity in Roquin^san/san^ mice that are homozygous for a point mutation in Rc3h1, the gene that encodes Roquin^17,18^. Indeed, Roquin^san/san^ mice showed dysregulation of immune response and used as a murine model of SLE^16,19^.

Thus, we hypothesized that IL-17 depletion would ameliorate the lupus-like characteristics of Roquin^san/san^ mice. Thus, we investigated the effect of loss of IL-17 on Tfh cells, GC formation, autoAb production, numbers of IL-17–producing T and B cells, and nephritis in Roquin^san/san^ and Roquin^san/san/^IL-17^−/−^ mice.

## Results

### AutoAb production and numbers of IL-17–producing T and B cells are increased in Roquin^san/san^ mice

**Figure 1:**
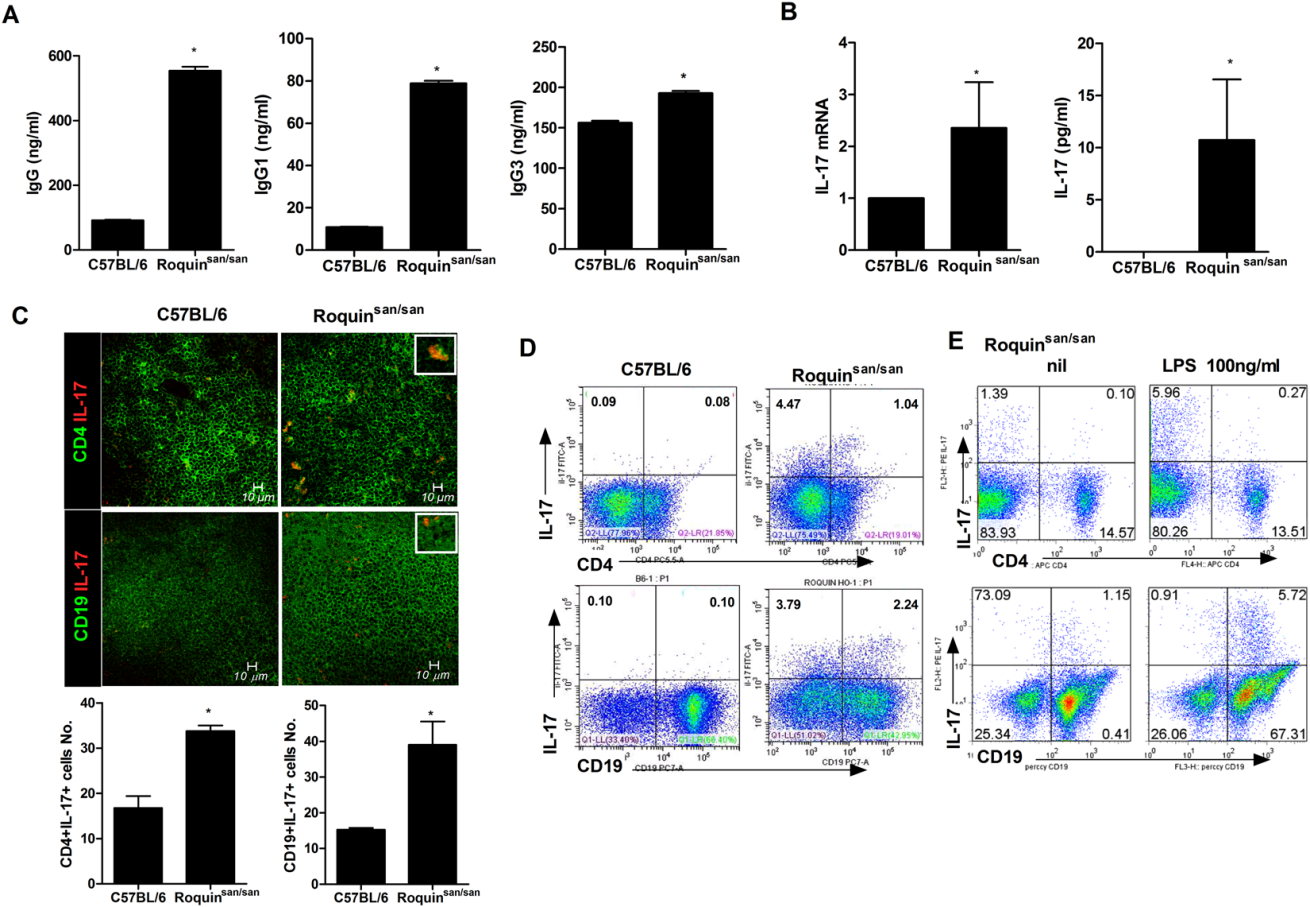
Immunoglobulin (Ig) production and the numbers of interleukin (IL)-17-producing T and B cells are increased in splenocytes and serum from Roquin^san/san^ mice compared to C57BL/6 and Roquin^san/san/IL-17−/−^ mice. (**A**,**B**) Serum IgG, IgG1, IgG3, and IL-17 levels were measured by enzyme-linked immunosorbent assay (ELISA) (each group n = 9). (**B**) IL-17 expression in splenocytes was determined by real-time PCR. (**C**,**D**) Confocal micrographs (n = 3). The numbers of CD4^+^ IL-17^+^ T cells and CD19^+^ IL-17^+^ B cells were determined by confocal microscopy and flow cytometry. (**E**) Splenocytes were simulated with lipopolysaccharide (LPS) (100 ng/mL) for 3 days and IL-17^+^ CD4 T cells and CD19 B cells were enumerated by flow cytometry. The numbers of CD4+ IL-17^+^ T cells and CD19^+^IL-17^+^ B cells were significantly increased in Roquin^san/san^ mice. Original magnification × 400. ^*^p < 0.05.

To investigate the potential function of Roquin on immune response, we used mouse genetics to mutant the Rc3h gene (Supplementary Fig. 1). We observed that IgG, IgG1, and IgG3 levels were increased significantly in Roquin^san/san^ mice compared to C57BL/6 mice (Fig. 1A). Moreover, the expression and production of IL-17 were upregulated significantly in Roquin^san/san^ mice compared to C57BL/6 mice (Fig. 1B). The numbers of IL-17–producing CD4^+^ T cells and CD19^+^ B cells were increased in Roquin^san/san^ mice (Fig. 1C,D). The frequency of IL-17–producing CD4^+^ T cells and CD19^+^ B cells in Roquin^san/san^ mice was increased by LPS treatment (Fig. 1E). These results suggest that the immune response in Roquin^san/san^ mice was enhanced by upregulation of IL-17 expression in T and B cells.

### Ig production and nephritis in Roquin^san/san/^IL-17^−/−^ mice

**Figure 2:**
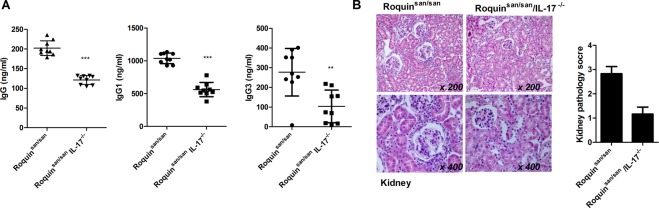
Ig production and nephritis in Roquin^san/san/^IL-17^−/−^ mice. (**A**) Serum IgG, IgG1, IgG3, and IL-17 levels in Roquin^san/san^ and Roquin^san/san/^IL-17^−/−^ mice (n = 9) were measured by ELISA. (**B**) Hematoxylin and eosin (H&E)-stained kidney sections from 15-week-old mice. The severity of the kidney pathology score was graded (n = 3). Original magnification × 200 and × 400. ^**^p < 0.01, ^***^p < 0.001.

Since we observed the upregulation of IL-17 level in Roquin^san/san^ mice, we hypothesized that IL-17 deficiency can reduce immune inflammatory response and SLE development. We used mouse genetics to mutant the Rc3h gene and IL-17a gene deficiency (Supplementary Fig. 1). Compared to Roquin^san/san^ mice, serum IgG, IgG1, and IgG3 levels were reduced significantly in Roquin^san/san^/IL-17^−/−^ mice (Fig. 2A). Nephritis was attenuated by IL-17 deficiency in Roquin^san/san^ mice (Fig. 2B). Therefore, IL-17 likely plays an important role in dysregulated humoral immunity in SLE.

### Treg cell differentiation is promoted by IL-17 deficiency in Roquin^san/san^ mice

**Figure 3:**
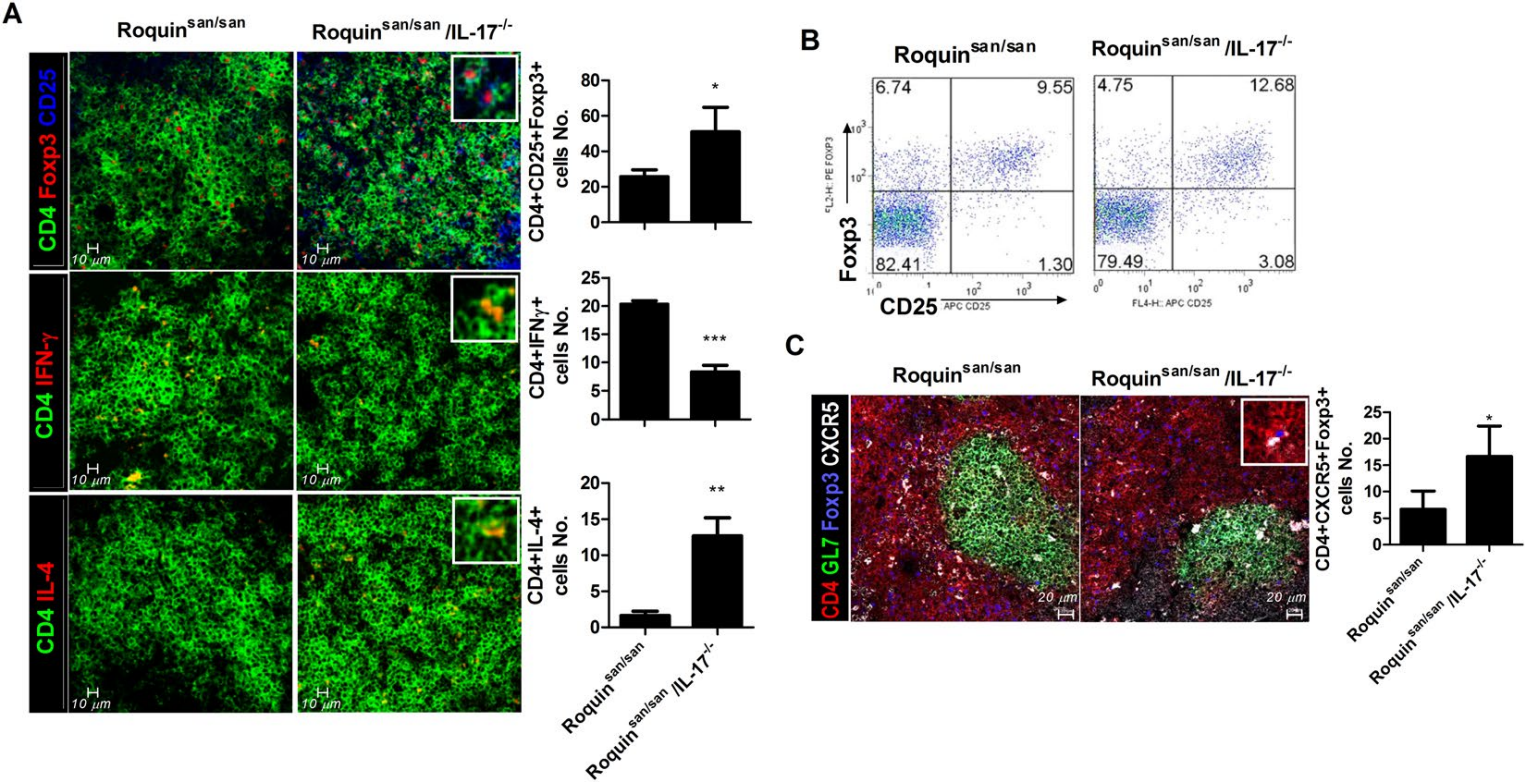
Effect of IL-17 deficiency on T-cell subsets in *Roquin*^*san/san*^ mice (n = 3). (**A**,**B**) The numbers of Foxp3^+^ regulatory T (Treg) cells, interferon (IFN)-γ^+^ Th1 cells, and IL-4^+^ Th2 cells were analyzed by confocal microscopy; representative images and a bar chart (right) are shown (Original magnification × 200). (**C**) The numbers of T helper (Th) cells and Tregs among splenocytes were determined by confocal microscopy (Original magnification × 400). ^**^p < 0.01, ^***^p < 0.001.

The number of Treg cells was increased significantly in Roquin^san/san/^IL-17^−/−^ mice compared to Roquin^san/san^ mice. Moreover, Th2 cell differentiation was increased, but Th17 cell differentiation was reduced significantly, in Roquin^san/san/^IL-17^−/−^ mice compared to Roquin^san/san^ mice (Fig. 3A). The frequency of Treg cells was confirmed by flow cytometry (Fig. 3B). Foxp3 expression in Tfh cells was also increased by IL-17 deficiency in Roquin mice (Fig. 3C). These findings suggest that loss of IL-17 results in an increased Treg population in Roquin^san/san^ mice.

### IL-17 deficiency inhibits Tfh cell differentiation in Roquin^san/san^ mice

**Figure 4:**
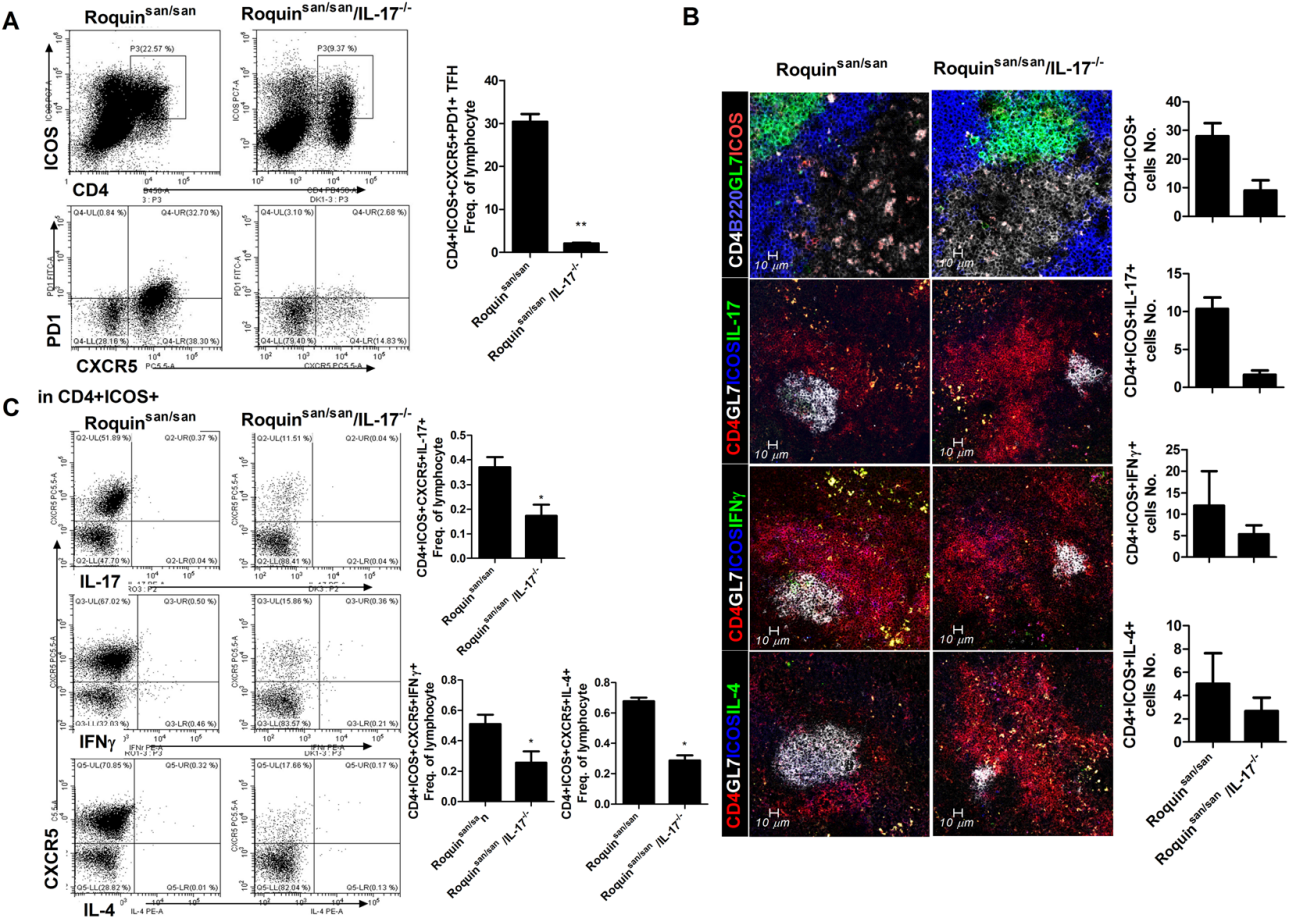
Effect of IL-17 deficiency on cytokine-producing Tfh cells in Roquin^san/san^ mice (n = 3). The numbers of CD4^+^ICOS^+^ CXCR5^+^PD1^+^ T follicular helper (Tfh) (**A**) cells and IL-17-, IFN-γ-, and IL-4-producing Tfh cells were analyzed by confocal microscopy. (**B**) Representative confocal micrographs of each mouse (n = 3). (**C**) IL-17^+^ Tfh, IFN-γ^+^ Tfh, and IL-4^+^ Tfh cells among splenocytes were analyzed by flow cytometry. Original magnification × 200. ^*^p < 0.05.

Tfh cells regulate the differentiation of B cells into plasma cells, which are involved in the pathogenesis of SLE. Therefore, we investigated the effect of IL-17 deficiency in Roquin^san/san^ mice on the numbers of cytokine-producing Tfh cells. The number of CD4^+^ICOS^+^ CXCR5^+^PD1^+^Tfh cells within the GC area was decreased in Roquin^san/san^/IL-17^−/−^ mice (Fig. 4A). The numbers of IL-17-, IFN-γ-, and IL-4-producing Tfh cells were determined by confocal microscopy and flow cytometry (Fig. 4B,C). These findings suggest that loss of IL-17 reduces the numbers of cytokine-producing Tfh cells in Roquin^san/san^ mice.

### IL-17 deficiency suppresses B-cell differentiation and GC formation in Roquin^san/san^ mice

**Figure 5:**
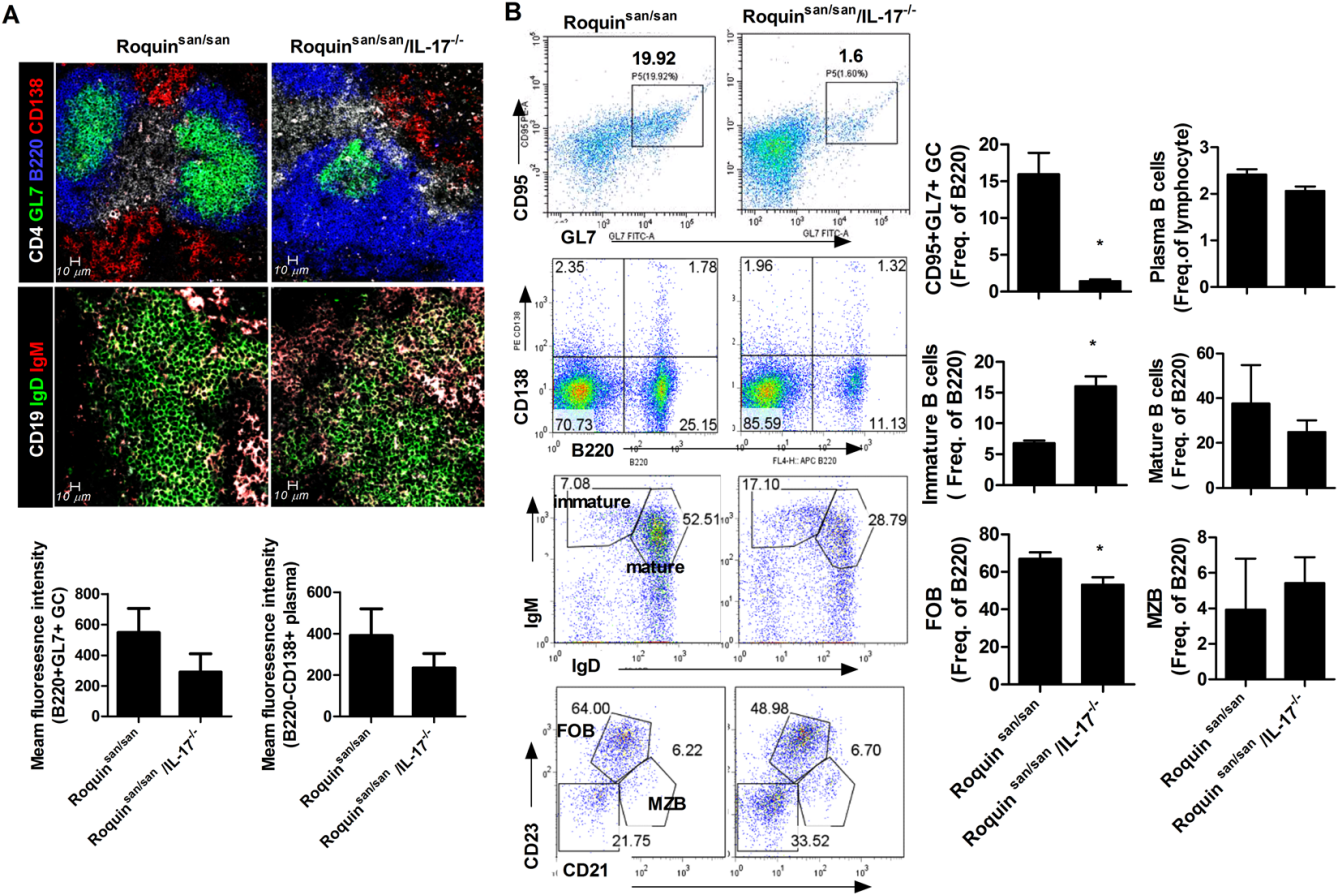
Effect of IL-17 deficiency on B-cell differentiation and germinal center (GC) formation in the spleen of Roquin^san/san^ mice (n = 3). (**A**) The numbers of B220^-^CD138^+^ plasma cells, B220^+^IgM^+^ immature B cells, and B220^+^IgD^+^ mature B cells were analyzed by confocal microscopy (Original magnification × 200 and × 400). (**B**) The numbers of plasma cells, immature B cells, mature B cells, CD21^high^CD23^low^ MZB cells, and CD21^mid^CD23^high^ FOB cells were determined by flow cytometry. IL-17 deficiency resulted in a significantly increased number of immature B cells in Roquin^san/san^ mice. ^*^p < 0.05.

GC formation and plasma cell activation are hallmarks of SLE. Therefore, we investigated the effect of IL-17 deficiency on B cell differentiation and GC formation in spleen tissue from Roquin^san/san^ mice. GC formation, CD138^+^ plasma cell, and CD19^+^IgD^+^ mature B cell differentiation were reduced in Roquin^san/san^/IL-17^−/−^ mice, while formation of CD19^+^IgM^+^ immature B cells was increased (Fig. 5A). The numbers of B cells, GC, plasma cells, immature B cells, and mature B cells were determined by flow cytometry (Fig. 5B). These results suggest that IL-17 deficiency in Roquin^san/san^ mice influences B-cell populations and inhibits the differentiation of pathogenic plasma cells and mature B cells.

### Effect of IL-17 deficiency on regulatory B cells in the spleen of Roquin^san/san^ mice

**Figure 6:**
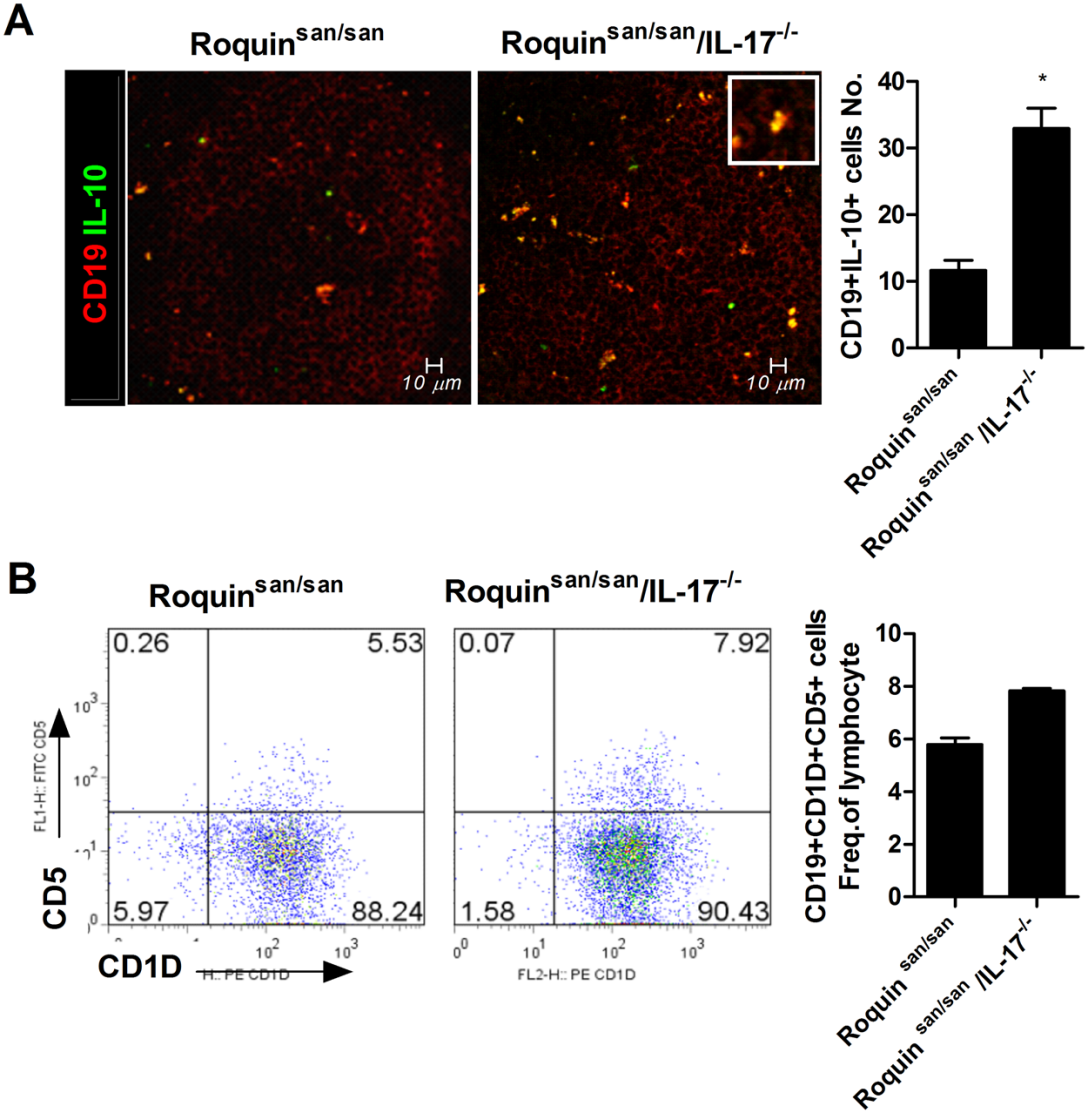
Effect of IL-17 deficiency on the numbers of IL-10-producing B cells and CD19^+^CD1d^+^CD5^+^ regulatory B cells (Bregs) in the spleen of Roquin^san/san^ mice (n = 3). (**A**) IL-10-producing B cells and Foxp3+ Bregs were analyzed by confocal microscopy. The number of IL-10-producing B cells was significantly increased in Roquin^san/san/^IL-17^−/−^ mice. (**B**) The number of CD19+ CD1d+ CD5+ regulatory Bregs as determined by flow cytometry. Original magnification × 400. ^*^p < 0.05.

The absence of regulatory B cells (Bregs) exacerbates pathologic inflammatory responses in autoimmune diseases. Therefore, we investigated the numbers of Bregs in IL-17-deficient Roquin^san/san^ mice. The numbers of CD19^+^IL-10^+^ Breg cells and CD19^+^CD1d^+^CD5^+^ Breg cells were increased in IL-17–deficient Roquin^san/san^ mice (Fig. 6A,B). We have shown the existence of IL-17–producing B cells in Roquin^san/san^ mice. These results suggest that IL-17^+^ B cells and Breg cells exert opposite effects on the autoimmune response in SLE.

## Discussion

Although IL-17 is associated with the pathogenesis of SLE, and Roquin^san/san^ is related to the SLE phenotype, the relationship between IL-17 and Roquin^san/san^ is unclear. SLE is characterized by systemic inflammation and overproduction of proinflammatory cytokines, including IL-17^6–8^. However, little information is recognized about the interaction of IL-17 and Roquin^san/san^ in T and B cells. Our results suggest that IL-17 deficiency in Roquin^san/san^ mice results in an increased number of IL-17-producing T and B cells, improvement of nephritis, and amelioration of the inflammatory response. Moreover, Roquin^san/san^ induced IL-17 expression in T and B cells. To our knowledge, this is the first report of increased IL-17 expression in T and B cells from Roquin^san/san^ mice. On the other hand, loss of IL-17 in Roquin^san/san^ mice ameliorated nephritis that is characteristic of SLE. Notably, IL-17 deficiency reduced the severity of inflammation in Roquin^san/san^ mice. This observation can elucidate IL-17 function related with inappropriate immune inflammation in SLE.

Dysregulation of IL-17 and Tfh cells is related to the pathogenesis of SLE. Differentiation of IL-17-producing Tfh cells was enhanced in BXD2 mice, which may be related to the development of SLE^20^. Moreover, Roquin^san/san^ mice exhibit an accumulation of Tfh cells and develop SLE^21,22^. Although suppression of IFN-γ production by Th1 cells reduces the severity of SLE in Roquin^san/san^ mice^23^, IL-17 expression in Tfh cells was not investigated. In this study, IL-17 deficiency in Roquin^san/san^ mice resulted in reduced numbers of IL-17-producing Tfh cells, leading to improvement of nephritis. These results suggest that IL-17 inhibition could be a therapeutic strategy in SLE.

AutoAb production and B-cell activation are related to the pathogenesis of SLE. B-cell activation leads to autoAb secretion^24^. Indeed, SLE is characterized by B-cell differentiation to plasma cells^25^. Moreover, the serum autoAb level is elevated in SLE patients^26^. B cell-targeted therapy involving inhibition of B-cell activation has been proposed^27^. In this study, IL-17 deficiency reduced B-cell differentiation and GC formation in Roquin^san/san^ mice. Serum IgG, IgG1, and IgG3 levels were decreased by IL-17 deficiency in Roquin^san/san^ mice. These results suggest that IL-17 has promise as a target for the development of novel therapeutics in SLE.

Because SLE is an inflammatory autoimmune disease^24^, Tregs and IL-10 are important factors in its treatment. Indeed, the frequency of CD4^+^CD25^high^FoxP3^+^ Tregs is decreased in SLE patients^28,29^. IL-10 is produced as an effector molecule by Tregs, and IL-10 receptor expression was reduced in a mouse model of SLE^30,31^. In this study, IL-17 deficiency enhanced Treg differentiation and IL-10 production by effector T cells in Roquin^san/san^ mice. Furthermore, IL-17 deficiency reduced the severity of SLE by increasing the number of Treg cells and the production of IL-10.

The effect of Roquin mutation on IL-17 production has to date been unclear. Our results provide insight into the role of IL-17 in the pathogenesis of SLE: Roquin mutation increased the expression of IL-17 in T and B cells. Thus, IL-17 can be considered a therapeutic target for SLE.

## Materials and Methods

### Ethics statement

The Animal Care Committee of The Catholic University of Korea approved the experimental protocol. All experimental procedures were evaluated and carried out in accordance with the protocols approved by the Animal Research Ethics Committee at the Catholic University of Korea (ID number:CUMC-2014-0103-03). All procedures performed followed the ethical guidelines on animal use.

### Animals

Male C57BL/6 mice (Jackson Laboratory, Bar Harbor, ME, USA), Roquin^san/san^ mice (Jackson Laboratory), and Roquin^san/san/^IL-17^−/−^ mice aged 15–20 weeks were maintained in groups (n = 9 per group) in polycarbonate cages in a specific pathogen-free environment. IL-17 KO mice were obtained from Dr. Y. Iwakura (University of Tokyo, Tokyo, Japan). Roquin^san/san^ mice were backcrossed with IL-17 knockout mice over 10 generations, and the mice were selected by genotyping PCR. The mice were provided with *ad libitum* access to mouse chow (Ralston Purina, St. Louis, MO, USA) and water.

### Measurement of immunoglobulin (Ig) and IL-17 concentrations

Serum concentrations of IgG, IgG1 and IgG3 were measured using mouse IgG, IgG1 and IgG3 enzyme-linked immunosorbent assay (ELISA) quantitation kits (Bethyl Laboratories, Montgomery, TX, USA). The serum IL-17 level was measured using an IL-17 DuoSet ELISA kit (R&D Systems, Minneapolis, MN, USA).

### Flow cytometry analysis of T, B and TFH cell populations

Splenocytes were isolated from the spleens of 15–20-week-old C57BL/6 and Roquin^san/san^ mice. B- and T-cell populations were identified using specific antibodies (eBioscience; San Diego, CA, USA). The 5 × 10^5^ T cells or B cells were stimulated for 4 h *ex vivo* with PMA (25 ng/ml, Sigma-Aldrich, St Louis, MO) and ionomycin (250 ng/ml, Sigma-Aldrich) in the presence of GolgiStop (BD Biosciences, Sparks, MD). To examine B-cell populations, splenocytes were stained with anti-B220- allophycocyanin (APC), anti-CD21-fluorescein isothiocyanate (FITC), anti-CD23- phycoerythrin (PE), anti-CD138-PE, anti-IgD-FITC, anti-IgM-PE, anti-CD1D-PE, anti- CD19-peridinin chlorophyll protein complex (PerCP), and anti-IL-17-PE antibodies. To analyze T helper and regulatory T (Treg) populations, splenocytes were stained with anti-CD4-PerCP, anti-IL-17-PE, anti-IL-4-PE, anti-interferon (IFN)-γ-FITC, anti-CD25-APC, and anti-Foxp3-PE antibodies. To analyze T follicular helper (TFH) cell populations, splenocytes were stained with anti-CD4-eFluor450, anti-CXCR5-PerCP-eFluor 710, anti-PD1-FTIC, anti-ICOS-PE cyanine7, anti-BCL6-APC, anti-IFN-γ-PE, anti-IL-4-PE, anti-IL-17-PE and anti-Foxp3-PE antibodies (Thermo Fisher Scientific, Waltham, MA). Flow cytometry was performed using a CytoFLEX flow cytometer (Beckman Coulter, IN, USA). The expressions of cell population were analyzed by CytExpert 2.3 software.

### Confocal microscopy

Spleen tissues were obtained from mice at 15–20 weeks after primary immunization, and the B- and T-cell populations of interest were identified using the following specific antibodies (eBioscience): anti-B220-APC, anti-GL7-FITC (or PerCP), anti-CXCR5-PerCP, anti-CD138-PE, anti-ICOS-PE (or APC), anti-IgD-FITC, anti-IgM-PE, anti-CD19-PE (or PerCP), anti-CD5-FITC, anti-IL-10-FITC, anti-Foxp3-PE, anti-CD4-PerCP (or FITC, PE), anti-CD25-APC, anti-Foxp3-FITC (or APC), anti-IL-17-PE, anti-IL-4-PE (or FITC), and anti-IFN-γ-FITC (or PE). Stained sections were visualized by confocal microscopy (LSM 510 Meta, Carl Zeiss, Oberkochen, Germany). The expression of GC or plasma B cells were estimated by comparing the mean fluorescence intensity using LSM 510 Meta, Carl Zeiss software.

### Immunohistopathological analysis of kidney

Mouse kidney tissues were fixed in 4% paraformaldehyde, decalcified in ethylenediaminetetraacetic acid (EDTA) bone decalcifier, and embedded in paraffin. Tissues were sectioned at 7 μm thickness, dewaxed using xylene, dehydrated through a gradient of alcohol, and stained with hematoxylin and eosin (H&E). The severity of the kidney pathology score was graded on a 0–4 scale as follows^32^: 0 = normal; 1, a small increase of cells in the glomerular mesanguim; 2, a larger number of cells in the mesangium; 3, glomerular lobular formation and thickened basement membrane; 4 glomerular crescent formation, sclerosis, tubular atrophy and casts.

### Statistical analysis

Statistical analysis was performed using IBM SPSS Statistics 20 for Windows (IBM Corp., Armonk, NY, USA). The significance of differences among multiple groups was evaluated by one-way analysis of variance (ANOVA); if a significant difference was detected, the Bonferroni post hoc test was used to assess the significance of differences between individual groups. Comparisons of numerical data were performed using the nonparametric Mann–Whitney *U* test (two-tailed). Values of *p* < 0.05 were considered indicative of statistical significance. Data are presented as means ± standard deviation (SD).

## Supplementary information

Supplementary Information
