# Supplementary figures and images for: Inhibition of IL-17 ameliorates systemic lupus erythematosus in Roquinsan/san mice through regulating the balance of TFH cells, GC B cells, Treg and Breg
Source: Sci Rep. 2019 Mar 26;9:5227. doi: 10.1038/s41598-019-41534-1 (PMC6435653; doi:10.1038/s41598-019-41534-1)

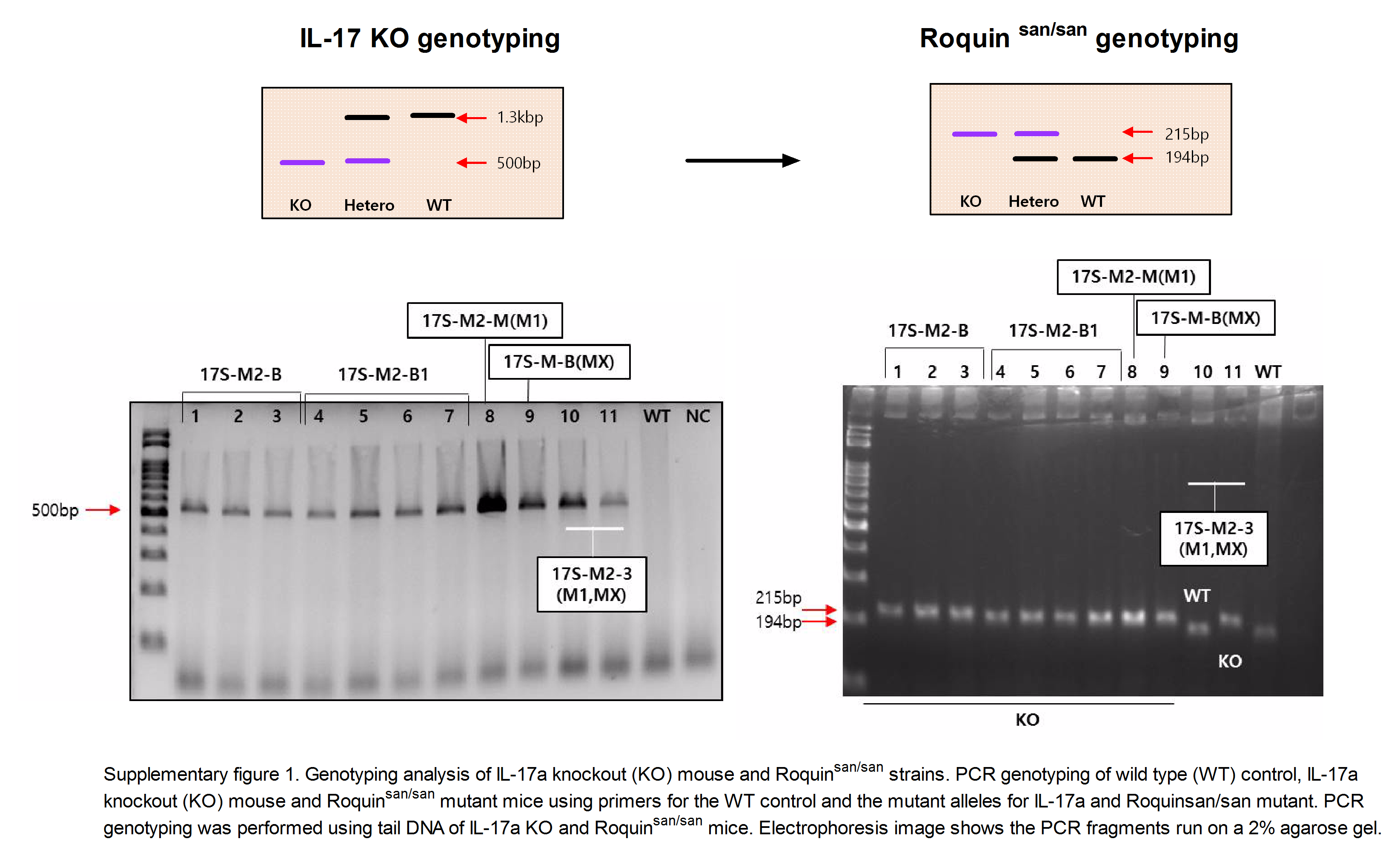

Supplement: Supplementary file 1 — Supplementary Information [file 41598_2019_41534_MOESM1_ESM.tif]
